# Representational specializations of the hippocampus in phylogenetic perspective

**DOI:** 10.1016/j.neulet.2017.04.065

**Published:** 2017-05-01

**Authors:** Elisabeth A. Murray, Steven P. Wise, Kim S. Graham

**Affiliations:** a Laboratory of Neuropsychology, NIMH, Building 49, Suite 1B80, 49 Convent Drive, Bethesda, MD 20892-4415, USA; b Olschefskie Institute for the Neurobiology of Knowledge, Potomac, MD 20854, USA; c Cognitive Neuroscience, School of Psychology, Cardiff University, CUBRIC Building, Maindy Road, Cardiff, CF24 4HQ, UK

**Keywords:** Anthropoid, Memory, Navigation, Perception, Representation, Scene memory

## Abstract

In a major evolutionary transition that occurred more than 520 million years ago, the earliest vertebrates adapted to a life of mobile, predatory foraging guided by distance receptors concentrated on their heads. Vision and olfaction served as the principal sensory systems for guiding their search for nutrients and safe haven. Among their neural innovations, these animals had a telencephalon that included a homologue of the hippocampus. Experiments on goldfish, turtles, lizards, rodents, macaque monkeys and humans have provided insight into the initial adaptive advantages provided by the hippocampus homologue. These findings indicate that it housed specialized map-like representations of odors and sights encountered at various locations in an animal’s home range, including the order and timing in which they should be encountered during a journey. Once these representations emerged in early vertebrates, they also enabled a variety of behaviors beyond navigation. In modern rodents and primates, for example, the specialized representations of the hippocampus enable the learning and performance of tasks involving serial order, timing, recency, relations, sequences of events and behavioral contexts. During primate evolution, certain aspects of these representations gained particular prominence, in part due to the advent of foveal vision in haplorhines. As anthropoid primates—the ancestors of monkeys, apes and humans—changed from small animals that foraged locally into large ones with an extensive home range, they made foraging choices at a distance based on visual scenes. Experimental evidence shows that the hippocampus of monkeys specializes in memories that reflect the representation of such scenes, rather than spatial processing in a general sense. Furthermore, and contrary to the idea that the hippocampus functions in memory to the exclusion of perception, brain imaging studies and lesion effects in humans show that its specialized representations support both the perception and memory of scenes and sequences.

## 1. Introduction

For out of olde feldes, as men seyth,
Cometh al this newe corn from yer to yere,
And out of olde bokes, in good feyth,
Cometh al this newe science that men lere.
Out of old fields comes all the new grain
Year after year, an old saying goes
And out of old books, new knowledge we gain
For people to learn, as everyone knows
—Parlement of Foules, Geoffrey Chaucer, 1382

Most experts agree that the hippocampus contributes to some of the most sophisticated aspects of human cognition, including episodic and autobiographical memory, scenario construction, constructive episodic simulation, future-thinking, prospection, perspective-taking and situational modeling. In contrast to these highly derived cognitive capacities, the evolution of the hippocampus can be traced to the earliest vertebrates. This combination of ancient ancestry and higher brain functions seems incongruous to some neuroscientists. Yet, as Chaucer recognized, it is sometimes the old that brings forth the new.

In this review we discuss the hippocampus and its homologues in a phylogenetic perspective. As we explain more fully in *The Evolution of Memory Systems: Ancestors, Anatomy, and Adaptations* [1], specialized representations emerged in the brains of particular ancestral species as they adapted to a new way of life, especially during major evolutionary transitions. Specific areas encode, process and store these representations, and the hippocampus homologue is one structure that does so. In early vertebrates it developed map-like representations that provided selective advantages for navigation. As new brain areas developed during subsequent evolution, the innovative representations in these areas influenced the hippocampus homologue and modified its function.

The human hippocampus performs many roles, but we focus here on its contribution to the perception and memory of visual scenes, with some comments on sequence memory. After a few points on evolution, we trace the modern form of this function to anthropoid adaptations in the Oligocene ~34 million years ago.

## 2. Evolution

### 2.1. Early vertebrates

**Fig. 1:**
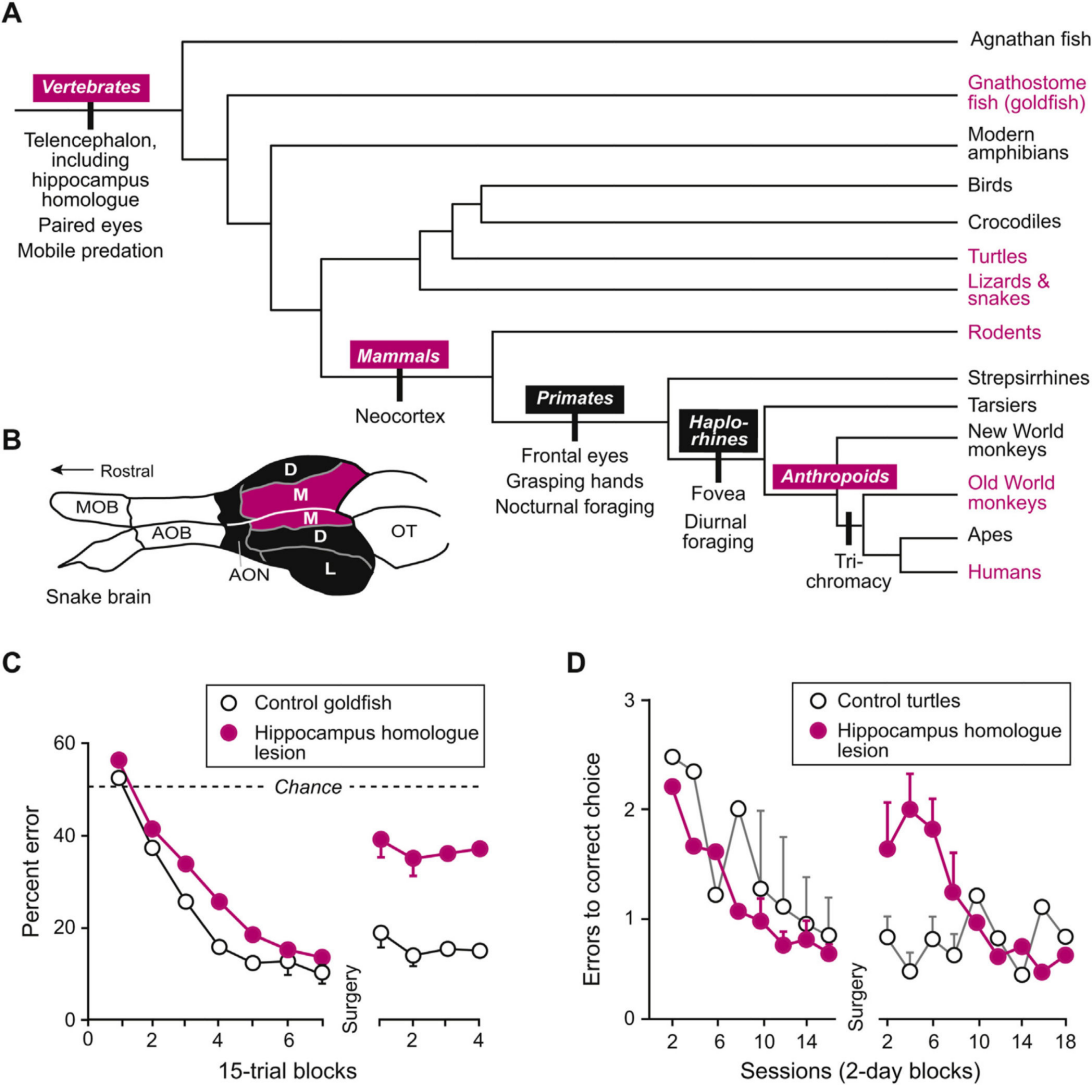
Vertebrate evolution and memory systems. (A) Cladogram designating selected lineages and some derived traits in those lineages. The magenta labels mark the animal groups highlighted in this article. (B) Drawing of a snake brain depicting three cortical fields: D, dorsal cortex; L, lateral cortex; M, medial cortex. The medial cortex is the reptilian homologue of the hippocampus. The snake brain is presented as representative of the brains of lizards, turtles and other non-avian, non-mammalian amniotes. Abbreviations: AOB, accessory olfactory bulb; AON, anterior olfactory nucleus; MOB, main olfactory bulb; OT, optic tectum. (C) Effect of removing the medial pallium, the everted homologue of the mammalian hippocampus, on maze learning and performance in goldfish [18]. Selected error bars show the standard error of the mean. (D) Effect of removing the medial cortex of turtles on their ability to navigate to a submerged platform containing food [19]. Format as in (C).

The major evolutionary transition that produced the first vertebrates occurred in the Cambrian or somewhat earlier [2]. These animals had a segmented musculature, a notochord, a dorsal nerve cord, paired eyes, olfactory organs on the head, and a brain that included the telencephalon, which consisted of a pallium and a subpallium. The hippocampus homologue emerged in early vertebrates as the medial pallium, a structure connected to the hypothalamus, septal nuclei, dorsal thalamus and lateral pallium [3–8]. The homologue of this structure is called the medial cortex in modern reptiles (Fig. 1B) and the hippocampus in mammals.

### 2.2. Anthropoids

As Fig. 1A illustrates, after the divergence of strepsirrhine and haplorhine primates, the latter split into tarsiers and anthropoids. Early haplorhines had switched from the nocturnal foraging of their ancestors to diurnal foraging, and they developed the primate fovea [9,10]. Anthropoids inherited both traits [11]. Over time, anthropoids increased in size: from the few hundred grams typical of the earliest species to several kilograms. The change in body size mostly occurred after the divergence of Old World and New World anthropoids in the early Oligocene, ~34 million years ago [12], and it required these animals to consume more nutrients than their smaller ancestors. As a result, anthropoid primates foraged over a larger home range than their ancestors had [13], and so they made foraging choices at a distance, based predominantly on the foveal vision that they inherited from their haplorhine ancestors. After Old World anthropoids developed the “routine” form of trichromatic vision, which requires a third opsin gene [12], color discrimination became particularly important. Their larger bodies and extensive home ranges favored a shift from leaping–grasping locomotion to the arboreal quadrupedal mode of traveling that enabled them to cover more territory. Of special interest to neuroscientists, these developments accompanied an upward grade-shift in brain size relative to body mass [14,15].

## 3. Functions of the hippocampus homologue

The hippocampus homologue of early vertebrates provided advantages in using vision and olfaction to guide behavior [1]. Collectively, the memory of sights and smells, their spatial layout, and the order and timing in which they should be encountered during a journey corresponds to a cognitive map [16], which enabled early vertebrates to navigate toward places that afforded either resources or safety. One important advantage of these map-like representations is that early vertebrates could use them to guide foraging and escape behaviors along novel routes. Another is that they could surmount unexpected obstacles. Their new capacities augmented an ancestral ability to navigate by responding to stimuli that they had encountered in the past. Put another way, representations in the hippocampus homologue enabled advantageous behaviors that transcended reflex-like responses reinforced by their past experience.

From a contemporary perspective, it is easy to underestimate how important navigation was to early vertebrates. Navigation is an important part of human behavior, of course—we would hardly have multimillion-dollar global positioning satellites otherwise—but it is only a limited aspect of human cognition. This was not the case for early vertebrates: Nearly all of their behavior involved navigation. Like many modern fishes, early vertebrates reproduced by depositing eggs in protected places and by dispersing sperm via navigation to these places. Likewise, these animals could only regulate their body temperature by moving to warmer or cooler locations. The parts of the hippocampus homologue nearest the amygdala became specialized for the control of diverse behaviors related to homeostatic, procreative, affective and autonomic functions [17], and the parts nearest the septal pole became specialized for the fine-grain analysis necessary for navigating within foraging fields and towards places of safety. This distinction should not be construed as a rigid dichotomy, of course. The effects of subtotal hippocampal lesions suggest gradations between these two classes of behavior, as opposed to strict compartmentalization.

Ample evidence shows that, among its other roles, the hippocampus homologue performs a conserved navigational function across modern vertebrates, including teleost fish, reptiles (*i.e.*, non-avian, non-mammalian amniotes), rodents, macaque monkeys and humans. The next five sections take up these topics, in turn.

### 3.1. Teleosts

Fig. 1C illustrates results from a maze-learning experiment in goldfish [18]. Like rodents, when goldfish start at novel places in a plus maze they can navigate to a remembered spatial goal. To do so, they need to make different movements relative to their body. For example, they might have to turn to the west rather than to their left at the choice point of the maze. And also like rodents, goldfish use novel routes and shortcuts that depend on map-like representations. These experiments also show that goldfish rely on integrated visual scenes rather than individual items in their field of view. As shown in Fig. 1C, lesions of the hippocampus homologue cause a significant impairment in navigating through the maze.

### 3.2. Reptiles

The medial cortex of reptiles plays a similar role, with the best evidence coming from studies of turtles [19] and lizards [20]. In both cases, lesions of the hippocampus homologue, labeled M in Fig. 1B, impair navigation. Fig. 1D illustrates results from a turtle-friendly version of the Morris water maze. In this experiment, four submerged platforms have food cups protruding above the surface. Only one of these cups has food, and the turtles learn to navigate to its location from a central starting point. Removal of the hippocampus homologue causes a significant impairment in returning to the correct platform [19]. After a few sessions, the turtles compensate for the lesion, and specific probe tests reveal how they do so. Rather than navigating via map-like representations, after the lesion they resort to snapshot memory, which involves recording the locations of particular items in the environment and later maneuvering to match current sensory inputs to these memories. Notably, their relearning might be aided by the use of a fixed starting point. When tested with novel starting points, the turtles with lesions of their hippocampal homologue perform poorly compared to control turtles.

In lizards, lesions of the hippocampus homologue cause an impairment in finding a warm rock among four rocks in a testing field [20]. Instead of navigating to the remembered warm rock as control lizards do, lizards with these lesions display thigmotaxis, hugging the border wall of the testing field much like rodents with hippocampus lesions do in the Morris water maze. Control lizards tend to use intramaze cues, unlike rodents in the Morris water maze, but this difference probably reflects the fact that lizards can see the rocks, in contrast to the invisible submerged platforms in the rodent experiments. This procedural difference also accounts for the finding that lesions of the dorsal cortex in lizards affects performance on the task. This area includes the homologue of the primary visual (striate) cortex of mammals [21], which probably provides visual information to the hippocampus homologue in intact (control) lizards.

### 3.3. Rodents

Our emphasis on an ancestral navigational function does not imply that the function of the hippocampus is limited to navigation. The same representations that support navigation can also contribute to a variety of other behaviors, including those that depend on timing, recency, relations, contexts, order and sequences.

**Fig. 2:**
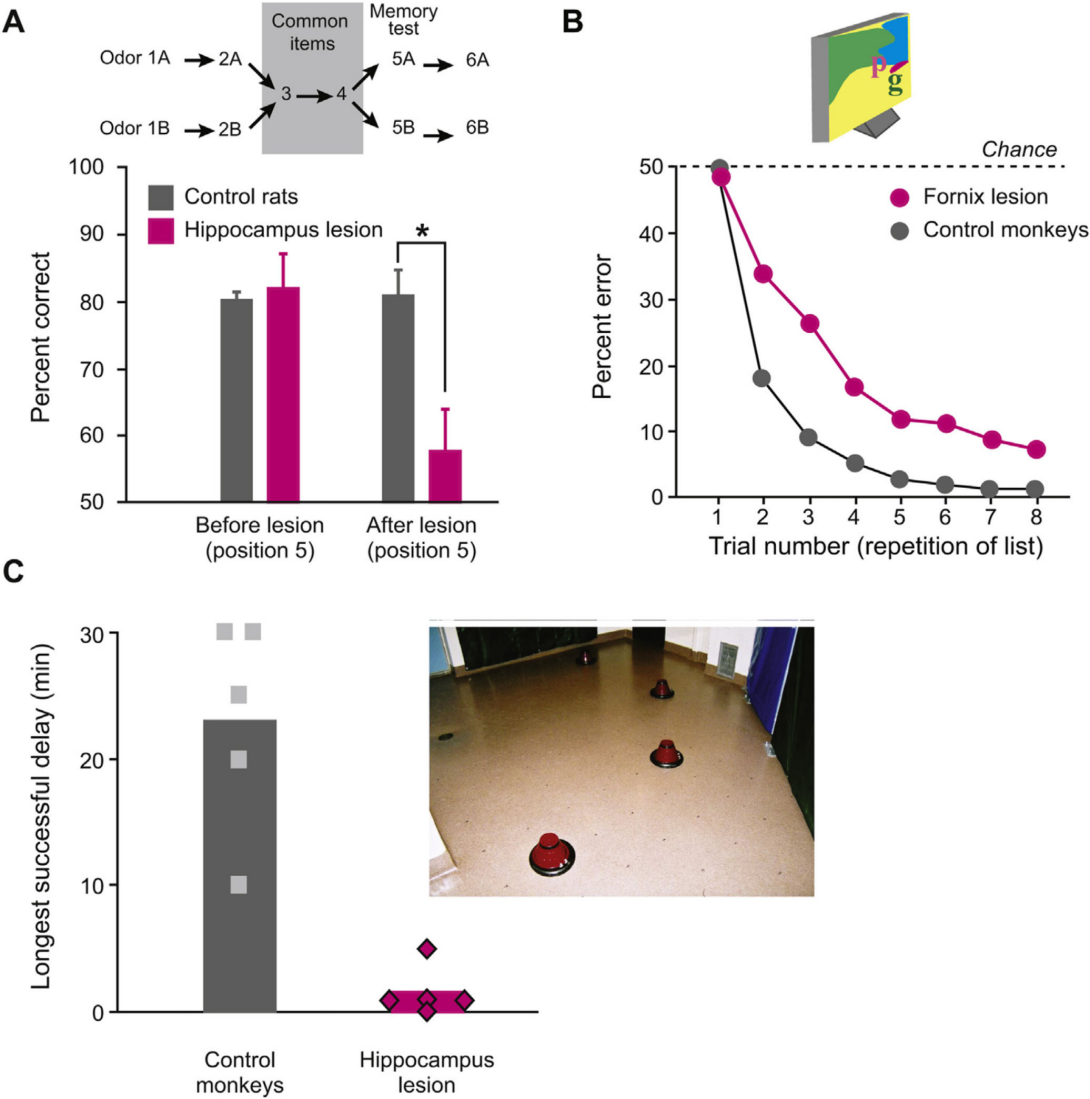
Effect of hippocampus or fornix lesions on task performance in monkeys and rodents. (A) Hippocampus lesions cause an impairment on an odor sequence task in rats [22]. Top: a depiction of two six-item sequences, which share elements at positions 3 and 4. After being presented with individual items 1A, 2A, 3 and 4, the rat faces a choice between odors 5A and 5B, with the former being correct. (B) Fornix lesions cause an impairment on the object-in-place scenes task in macaque monkeys [36]. (C) In monkeys, hippocampus lesions cause an impairment in remembering the location of food items hidden under inverted flower pots (inset) in an open field [38]. After successful performance, the length of the memory delay period was increased until the monkey could no longer perform the task at 83% correct or better. Symbols show the performance of individual subjects; bars show the group means.

Fig. 2A presents a single example selected from an extensive rodent literature [22]. The hippocampus plays a particularly important role in representing sequences, a capacity crucial for navigation and also essential for many laboratory tasks. In one experiment, rats learn two sequences of odors, both of which have six items and share items 3 and 4. In these experiments, the rats make choices among cups with odorous sand, which sometimes covers food. The memory tests begin by restricting the available cups so that the rats have to dig for food in the first two items of a given sequence. Next, they dig into the two cups shared by the two sequences, items 3 and 4. Finally, they face a choice between the fifth item of the two sequences. To obtain more food, they need to choose the odor that continues the correct sequence. Fig. 2A shows that rats with lesions of the hippocampus have a significant impairment on this task. They have no deficit on a control task that requires the detection and choice of novel odors.

The combination of sequences of stimuli and their timing define a navigational journey. The properties of place cells are well known, including their signaling of the starting and ending points of a journey [23], but cells in the hippocampus also encode timing information. Some of its neurons signal the relative time within a journey, and populations of such neurons encode the progress that a rat makes through a temporally extended series of events [24]. For example, in a sample and choice matching task, time-cell activity fills the delay period [25], and similar temporal signaling occurs as rats walk on a treadmill [26]. These properties have obvious relevance to navigation, but they also subserve recency, sequence and order memories. For example, when rats need to make a choice based on the relative recency of an odor, as it appeared in a sequence, activity in the hippocampus correlates with the accuracy of the choice [27].

The findings mentioned here come from a vast rodent literature, of course, and elsewhere [1] we discuss additional examples that point to a conserved navigational function of the hippocampus. This role depends on map-like spatiotemporal representations that encode the order and sequence of olfactory, visual and other stimuli encountered during a foraging journey or in finding safe haven. During the evolution of amniotes, these specialized representations became subject to selection and were affected by innovations elsewhere in the cerebral cortex.

### 3.4. Macaques

At first glance, little of the literature on the primate hippocampus seems related to navigation. Instead, neuropsychological research on macaque monkeys has focused either on a role in spatial memory or on developing a monkey model of explicit (declarative) memory. Taking their lead from rodent research, monkey neuropsychologists in the 1980s and 1990s concluded—wrongly as it turned out—that the macaque hippocampus subserves spatial memory in a general sense. Had they considered the life that these animals lead in their natural habitat, they might have come to a different conclusion.

Early investigations, for example, reported that hippocampus lesions cause impairments on the spatial reversal task [28,29], but this result depended on inadvertent damage to the nearby parahippocampal cortex and fiber pathways. Later studies show that lesions limited to the hippocampus do not affect performance on this task [30]. Likewise, Mishkin and his colleagues reported that hippocampus lesions impair performance on an object-in-place memory task [31,32]. Again, this false-positive result can be traced to inadvertent brain damage. Unlike selective lesions of the hippocampus, lesions of the parahippocampal cortex (which include parts of the subicular complex) yield impairments on this task [33]. A similar account likely applies to impairments reported for spatial matching-to-sample and nonmatching-to-sample tasks conducted with a manual test apparatus [34].

In all of these experiments, aspiration lesions of the hippocampus involved removal of both the subicular complex and the parahippocampal cortex, along with the caudal part of the entorhinal cortex. This surgical procedure provided a convenient way to gain access to the hippocampus, but it made the results difficult to interpret. When subsequent experiments employed selective lesions of the hippocampus, monkeys performed normally on the standard spatial memory tasks.

Instead of a general function in spatial memory, the macaque hippocampus plays a specific role in choices based on integrated background scenes. As explained in Section 2.2, distant background scenes correspond to the kinds of stimuli that anthropoids use for navigation to resources in their natural habitat, as their ancestors likely did in the Oligocene. Standard neuropsychological tests, such as the object-in-place task described above, do not depend on back-ground scenes. This task, for example, involves a single test tray with three fixed locations, each containing a single item. This problem lends itself to many possible solutions, mostly based on the individual items and their relationship to the tray’s geometry, to the monkey and to each other (e.g., landmarks, egocentric cues, etc.).

Fig. 2B shows the effect of fornix lesions on performance of the object-in-place scenes task. In the inset, the letter “p” serves as the correct target on a given background scene. Control monkeys learn to choose correctly after only a few trials with a given scene, interleaved with 19 other scenes and targets, but monkeys with fornix transections have a significant impairment [35–37]. These behaviors do not require navigation, but the kinds of neural representations required for this task would have obvious advantages in that domain. The rapid learning in control monkeys supports the idea that macaques are well adapted to learning about visual scenes. Note that—despite their similar names—the object-in-place task differs from the object-in-place scenes task in a crucial way: in the latter, a large, integrated background scene contributes to rapid learning.

Fig. 2C illustrates the effect of hippocampus lesions on an overtly navigational task in macaques. Formally, this task corresponds to a spatial matching-to-sample task. In this experiment, monkeys see a set of inverted flower pots in a foraging field, but only one of them covers food. In the sample presentation, the monkeys explore the flower pots until they find some food. After returning to their cage for a variable delay period, the monkeys can later return to that site, which once again contains food. As noted earlier, selective hippocampus lesions do not affect performance on traditional versions of the spatial matching-to-sample task, which involve reaching to a cued location on a test tray [34]. However, when the same kind of test occurs in an open foraging field, hippocampus lesions cause a significant impairment [38]. To tax navigational memory in these experiments, the memory interval increases gradually until each monkey performs at less than 83% correct. In this test, control (intact) monkeys usually meet this criterion with memory intervals of 20–30 min. In contrast, monkeys with lesions of the hippocampus fail to achieve this criterion at delays of only 2 min (Fig. 2C). Fornix lesions cause a similar impairment as macaque monkeys perform a spatial nonmatching-to-sample task in a T-maze [39]. Here, the monkeys navigate through the maze based on extramaze cues located in the testing room, which probably provide an integrated background scene to guide their choices. Other experiments in macaques resemble the studies in rodents that reveal a role for the hippocampus in order and sequence memory. For example, lesions of the fornix in macaque monkeys cause an impairment in making choices based on relative recency [40].

### 3.5. Humans

In accord with results from the object-in-place scenes task in monkeys, patients with lesions of the hippocampus have impairments in the perception and memory of visual scenes. This point has been demonstrated using a variety of tasks, including recognition memory [41,42], discrimination learning [43,44], and odd-stimulus-out (oddity) judgment [45] tasks. As reviewed previously [46–48], patients with lesions restricted to the hippocampus have a preserved capacity when tested on other kinds of visual stimuli, such as faces or dot patterns [44,49]. According to our proposal, the prominence of scene representations in the human hippocampus reflects an inheritance from Oligocene anthropoids, which used background scenes to make foraging choices at a distance.

**Fig. 3:**
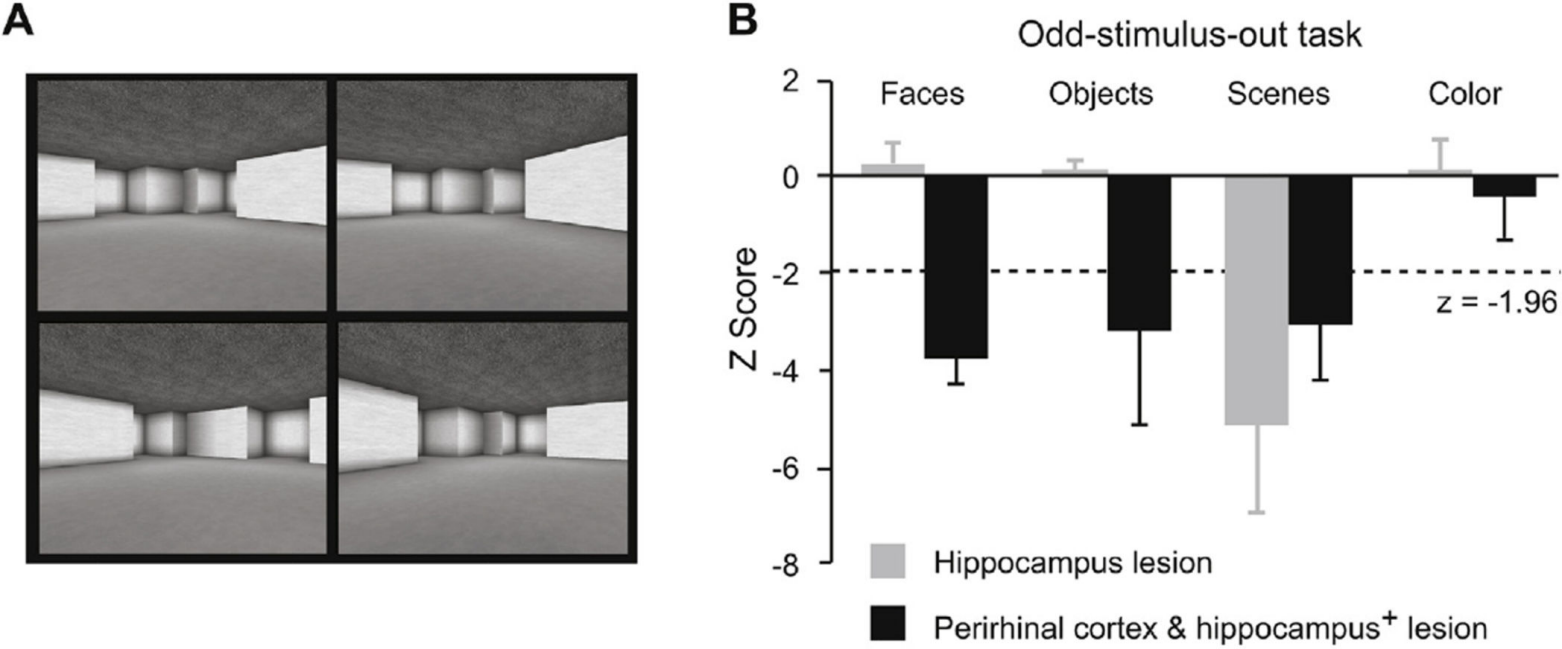
Specializations of the human hippocampus. (A) Scenes used in the odd-stimulus-out task. The lower, left scene is the odd one out. (B) Scores on the odd-stimulus-out task with four types of stimulus material. The dashed line shows the level of impairment that reached statistical significance. Error bars: standard error. The label “perirhinal cortex and hippocampus^+^” denotes that the lesion included additional parts of the temporal lobe. Plot from Lee et al. [46].

Fig. 3A illustrates a version of the odd-stimulus-out task using four visual scenes. The subject’s job is to identify the scene that differs from the other three. These experiments avoid the prior training that occurs in analogous monkey experiments, but the results are similar. Two kinds of patients are compared: those with lesions mainly confined to the hippocampus and those with larger lesions that include both the hippocampus and the perirhinal cortex, among other parts of the medial temporal lobe. Both kinds of patients perform well on standard tests of perception, but Fig. 3B shows that they both have a perceptual impairment for visual scenes. Restricted hippocampus lesions cause an impairment in identifying the odd-stimulus-out for pictures of scenes, but not for pictures of objects, faces, or patches of color. Patients with larger lesions of the medial temporal lobe have impairments for objects, scenes and faces, but not for colors. Note that the odd-stimulus-out task does not measure stimulus memory: All of the requisite information is available simultaneously and the stimuli are unique to each trial. Accordingly, these findings highlight the importance of the stimulus material used in testing, with crucial distinctions among different kinds of visual stimuli. When stimuli tax the specialized representations of the human hippocampus, lesions cause an impairment in both perception and memory.

**Fig. 4:**
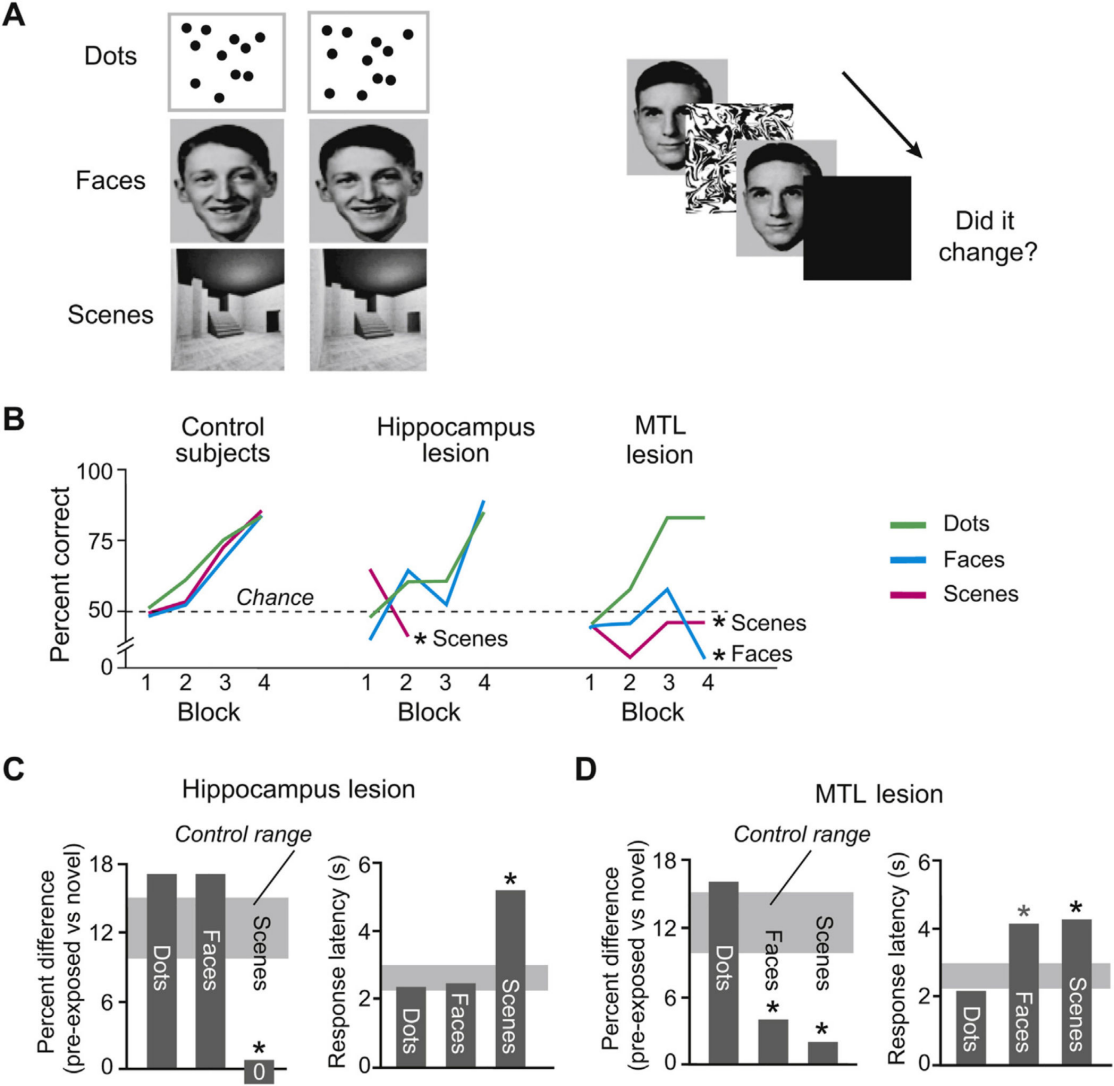
Visual discrimination for three types of stimulus material. (A) Examples of dot, face and scene stimuli. Participants indicate whether a pair of stimuli are the same or different. (B) Improvements in performance accuracy as a function of learning, across four blocks of trials. Asterisks indicate statistically significant impairments in learning in two patients. MTL: medial temporal lobe, with involvement of both the perirhinal cortex and hippocampus. (C, D) Pre-exposure to the stimuli leads to perceptual learning, which aids both performance accuracy and response latencies. “Percent difference” indicates the difference in accuracy on trials with novel vs. pre-exposed material. The larger the percent difference in performance accuracy and the shorter the response latency, the greater the advantage conferred by pre-exposure to the stimulus material. Range of performance (defined as the standard error of the mean) for control patients is shown by the gray shading. Asterisks indicate statistically significant differences from control performance. Data from Mundy et al. [44].

In a related experiment, perceptual learning leads to performance benefits [44]. Participants are pre-exposed to visual scenes, faces or dot patterns (Fig. 4A). They then see sequential pairs of images and have to indicate whether a current image differs from the previous one. A patient with a lesion restricted mainly to the hippocampus has an impairment in scene learning (Fig. 4B, middle, magenta line), but not in learning about faces (blue line) or dot patterns (green line). In this patient, pre-exposure to the stimulus material benefits both the accuracy of performance (Fig. 4C, left) and response latencies (Fig. 4C, right) for faces and dot patterns, but not for scenes. In a patient whose lesion included more of the medial temporal lobe, including the perirhinal cortex, an impairment is evident for the learning of both scenes and faces, but not of dot patterns (Fig. 4B, right, blue and magenta lines; Fig. 4D). Fig. 4B (left) and the gray shading in Fig. 4C and D show that—in both of these patients—whenever their lesions spare performance for a certain kind of stimulus material (*e.g*., dot patterns), they perform as well or better than control participants.

**Fig. 5:**
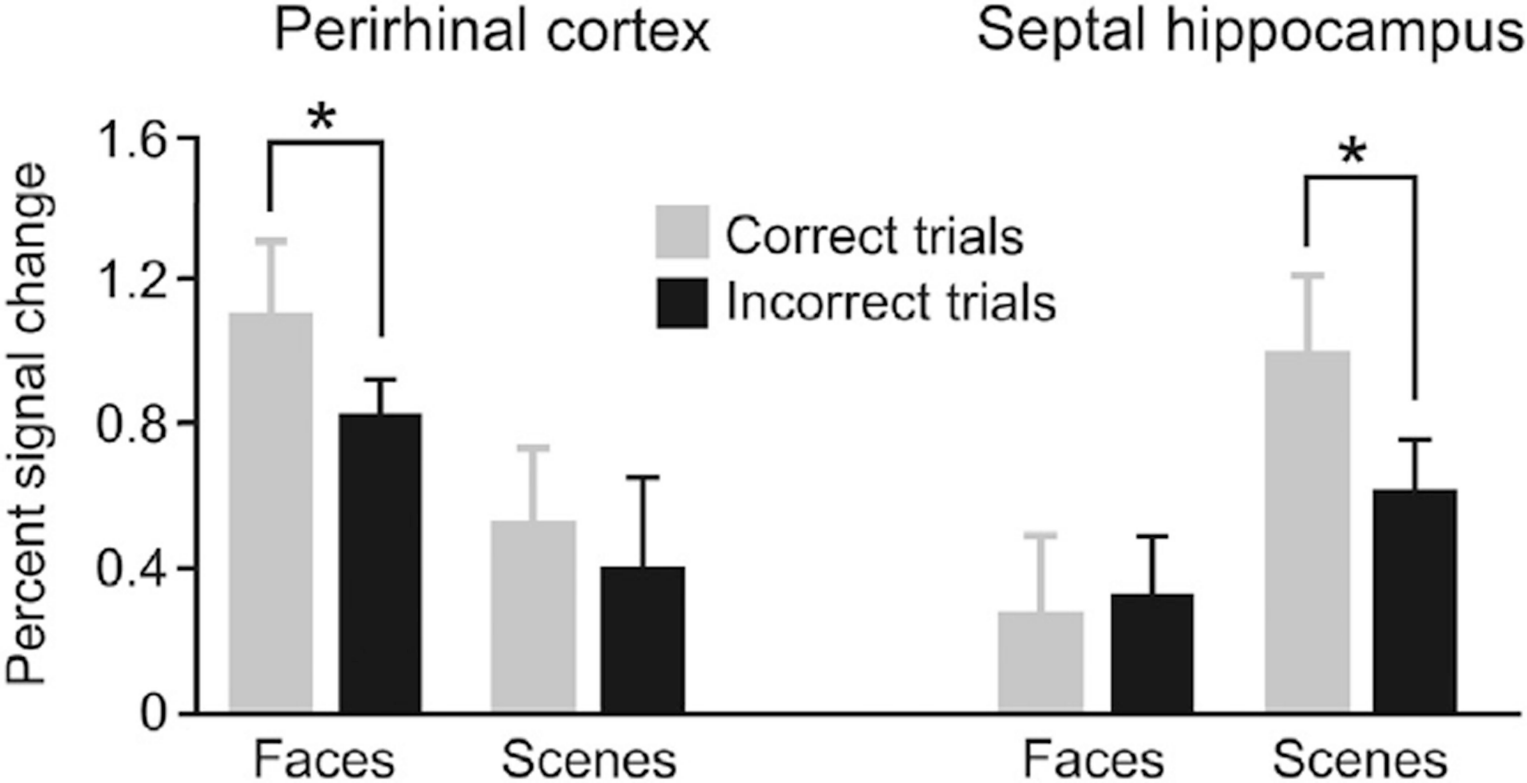
Brain imaging activations for the faces and scenes tasks illustrated in Fig. 4A. Percent signal change for correctly versus incorrectly discriminated trials. The asterisks indicate statistically significant differences. Error bars: standard error. Data from Mundy et al. [44].

Fig. 5 displays complementary results from a functional neuroimaging experiment [44]. In a contrast between correctly and incorrectly performed trials, activations in the hippocampus show a difference for scenes but not faces (Fig. 5, right), and those in the perirhinal cortex have the opposite pattern (Fig. 5, left). Likewise, multivoxel decoding of activations in the hippocampus can detect scene discriminations and not object discriminations [50], with the opposite results in the perirhinal cortex [51]. A similar result comes from a viewpoint-discrimination task for scenes, one based on the same odd-stimulus-out design mentioned earlier [52]. Performance on this task is affected by damage to the hippocampus, and viewpoint judgements are associated with hippocampus activation for scenes. In contrast, the perirhinal cortex shows such activation for objects, and damage to this structure, alongside hippocampus injury, results in impairments on the odd-stimulus-out task for both scenes and objects [52]. Other studies show that these functional specializations not only relate to these cortical areas, but also to the key white matter pathways conveying the main inputs and outputs to them [53,54]. Finally, visually presented words that describe scenes activate the anterior hippocampus more than words about objects or abstractions, in accord with the impairments reported for patients with bilateral hippocampus damage in constructing imaginary scenes [55].

Although standard perception tests have consistently failed to reveal impairments after hippocampus damage, they do not tax the specific kinds of representations that evolved in the hippocampus. Tests such as complex drawing, for example, can reflect the perception of features and not integrated scenes. The perception of dot patterns and letters draw on integrated visual features but not on the specific kinds of integrated scenes that the hippocampus represents. In contrast to theories that posit segregated cortical areas for perception and memory, the evidence reviewed here indicates that specialized representations in the hippocampus support both the perception and memory of visual scenes. The hippocampus not only plays a role in the *explicit* perception of scenes, but also in implicit scene perception [56,57], implicit scene memory [58] and perceptual learning about scenes (Fig. 4) [47,59]. Furthermore, a component of the hippocampus, the dentate gyrus, is thought to play a role in pattern separation for scenes [60], among other representations, and this probably applies to both explicit and implicit discrimination.

Of course, we do not mean to imply that the hippocampus works alone for any of these capacities. The hippocampus is only one part of a broader scene processing network, which includes the subiculum [61], posterior parahippocampal gyrus, retrosplenial cortex and transverse occipital sulcus [62]. Scene analysis also draws on representations in the entorhinal cortex, where grid-cell activity (in rodents and macaques) and grid-cell-like activations (in humans) signal position and navigational progress [63–65].

Finally, notwithstanding our emphasis on visual scenes—and therefore our inheritance from Oligocene anthropoids—representations in the hippocampus support a broad variety of behaviors related to navigation. For rodents (Section 3.3) we highlighted sequence learning; for macaque monkeys (Section 3.4) we mentioned a role in recency judgements. For humans, recent neuroimaging research has shown that the pattern of activation in the human hippocampus carries information about the serial order of items in learned sequences but not about items or order in random sequences [66–68].

In one experimental design, participants see several sequences, each consisting of five images of familiar objects, animals, fruits, vegetables or other items [68]. Fixed sequences consist of five items presented repeatedly in a given order, including unique sequences and those with shared items like the sequence illustrated in Fig. 2A; other sequences involve items presented in a random order. After the learning phase of the experiment, a multivoxel pattern analysis shows that activation patterns in the hippocampus carry information about the conjunction of an item and its position in an ordered sequence, as contrasted with information solely about an item or a sequential position. Participants with stronger encoding of item-position conjunctions also have faster reaction times for semantic judgments about the items. Because decoding does not reveal item-order conjunctions in either the perirhinal or parahippocampal cortex, these results appear to reflect conjunctive representations specific to the hippocampus.

## 4. Conclusions

A homologue of the hippocampus emerged during the evolution of early vertebrates, and it specialized in representations that provided an advantage in navigation. During anthropoid evolution, the representation of visual background scenes became important for their foraging choices, especially at a distance. The conserved representations of the hippocampus also contribute to tasks that require recency, sequence, timing and order judgments, and evolving humans inherited all of these traits. As a result, representations in the human hippocampus support the perception of scenes, perceptual learning about scenes, and both explicit and implicit memories about scenes. In *The Evolution of Memory Systems* [1], we explore how these representations also serve as the basis for some additional cognitive capacities, such as episodic and autobiographical memory, perspective-taking and constructive episodic simulation.

## HIGHLIGHTS

- A homologue of the hippocampus emerged in early vertebrates.
- It provided these mobile ancestors with representations that supported navigation.
- These representations also enabled non-navigational functions, *e.g*., serial order.
- Among primates, anthropoids came to use foveal vision for foraging at a distance.
- This trait favored representations for perceiving and remembering visual scenes.
